# Post-discharge Mortality in Patients With Delirium and Dementia: A 3-Year Follow Up Study

**DOI:** 10.3389/fpsyt.2022.835696

**Published:** 2022-02-28

**Authors:** Thiemo Schnorr, Tim Fleiner, Henning Schroeder, Ira Reupke, Frank Woringen, Rieke Trumpf, Stefan Schroeder, Wiebren Zijlstra, Peter Haussermann

**Affiliations:** ^1^Department of Geriatric Psychiatry and Psychotherapy, LVR-Hospital Cologne, Cologne, Germany; ^2^Institute of Movement and Sport Gerontology, German Sport University Cologne, Cologne, Germany; ^3^Department of Psychiatry, KMG Kliniken, Güstrow, Germany; ^4^Brandenburg Medical School Theodor Fontane, Neuruppin, Germany

**Keywords:** dementia, delirium, aftercare, mortality, clinical research

## Abstract

**Background:**

Delirium and dementia are prominent psychiatric diseases in old age and connected with poor outcomes for people affected. Nevertheless, there is a lack of knowledge concerning the long-term prognosis of patients with dementia and delirium. This study analyzes mortality, readmission rates and discharge destinations of patients with dementia or delirium superimposed on dementia (DSD) within 3 years after discharge from hospital.

**Methods:**

A cross-sectional, monocentric cohort study was conducted at the department of geriatric psychiatry of the LVR hospital cologne, using structured telephone interviews and analyses from the clinical information system. All patients with dementia and DSD, admitted between December 2014 and November 2015, were screened for eligibility.

**Results:**

In total, 113 patients were included, 49 patients with dementia (*M* 80 years, female 49%) and 64 with DSD (*M* 82 years, female 47%). Three years after discharge, 66 patients (58%) had died (95% CI 91.9–112.5; *p* = 0.53). Within the first 3 months, 9 patients (14%) with DSD deceased, but no patient from the dementia group (95% CI 11.3–12.7; *p* = 0.01). Out of all patients, 17 patients were readmitted and nursing homes were the predominant discharge destination (55%).

**Conclusions:**

This analysis revealed a high post-discharge mortality rate of patients with dementia and DSD. For patients with DSD, a close clinical monitoring, mainly within the first 3 months after discharge, should challenge the significantly increased acute-mortality. These findings should set the pattern for a comprehensive analysis of long-term effects of dementia and DSD. More studies are required for better understanding and comparability in this field of research and healthcare.

## Introduction

The hospital treatment of patients with dementia is often caused by or complicated due to the occurrence of delirium and neuropsychiatric symptoms (1). Delirium is one of the most common complications in hospitalized older adults, with a prevalence of between 14 and 64% and hospital mortality rates from 25 to 33% (2, 3). Neuropsychiatric symptoms occur in almost every patient with dementia (4). The multidisciplinary goal of hospital treatment in special dementia care units is to facilitate recovery and to provide the patient as well as their caregivers with a discharge to the best fitting destination. Besides the increasing evidence on individualized treatment in acute care, there is a paucity of studies analyzing long-term follow-up of inpatient care. Until now, few investigations have focused on the aftercare of patients with dementia and/or delirium (5). Dementia alone, delirium alone and delirium superimposed on dementia (DSD) are associated with an increased risk of mortality in geriatric acute care and in long-term analysis after discharge (6). The largest follow-up analysis until now is from the United Kingdom, including 6,724 patients aged ≥ 65 years, reporting that 53% of the patients with cognitive impairment died within 2 years after admission to hospital care (7). The highest mortality risk for delirium alone was reported after 6 months and after 1 year; in the medium- to long-term analysis patients with dementia showed higher mortality rates (7). A Brazilian investigation including geriatric patients reported significant differences after hospital discharge, whereas 50% of patients with delirium and 34% of patients without delirium died within the first year after discharge (8). Patients with dementia are at high risk of readmission within 30 days after discharge (9). To the best of our knowledge, no analysis of long-term readmissions in this group has been conducted yet. An analysis of discharge destinations from acute psychogeriatric wards in North-West England reported an ability to return to the previous setting in 92 patients (78%) and a discharge to another setting in 26 patients (22%), with 82 patients (83%) being able to return to home (10). Adequate treatment of delirium and dementia is an important issue in acute care; however, there is a lack of research on long-term effects and discharge management in acute mental health care (10). Therefore, the aim of this study is to investigate mortality in a follow-up analysis after discharge from acute care of patients with delirium and dementia as well as readmissions and aftercare.

## Methods

### Study Design and Sample

A cross-sectional study of the patients admitted to the department of geriatric psychiatry at the LVR-Hospital Cologne, Germany, from December 1st, 2014 until November 30th, 2015 was conducted. The geriatric psychiatry department includes secured and open wards and provides acute care for geriatric patients with psychiatric disorders. Admitted patients mostly experience an acute risk to their health or are at acute danger to harm themselves or others. Patient's discharge will be realized as soon as the health status is stabilized (e.g., remission of delirium). In the German health system, patients with delirium will be admitted to a geriatric psychiatry ward when the underlying medical causes were stabilized but behavioral disturbances, such as aggression and disorientation, or cognitive disturbances are still present. All procedures involving human subjects/patients were approved by the Ethics Committee of the North Rhine Medical Association Chamber (reference number 2018192). The trial registration number is DRKS00006740 (German Clinical Trials Register). All patients with a diagnosis of dementia or delirium admitted to the department of geriatric psychiatry were screened for eligibility for this study. Written informed consent was obtained from all included patients (respectively their legal guardian). Cases were included on the following criteria: (1) age 60 or higher, (2) diagnosis of dementia or DSD following the International Classification of Diseases 10th Revision (ICD-10) criteria (11), and (3) written consent of the legal guardian. Exclusion criteria were: (1) incomplete data on main outcome, and (2) no consent to participate. Participants were allocated to the dementia only and the DSD group by their ICD-10-diagnosis.

### Instruments

The patient's characteristics were derived from the standardized comprehensive geriatric assessment, conducted within the first 24 h after admission. Data included age, sex, body mass index (BMI), diagnosis according to the ICD-10 criteria (11), Mini-Mental State Examination (MMSE) score, Bayer activities of daily living (B-ADL) score and living status at admission, extracted from the clinical hospital information system for the participants of this study. The validated German version of the confusion assessment method (CAM) was conducted in order to screen for delirium (12, 13). The CAM is a proxy assessment with four items: acute onset and fluctuating course, inattention, disorganized thinking, and altered level of consciousness. The MMSE is a validated assessment for cognitive impairment in patients with dementia (14). It includes 30 questions or tasks concerning basic cognitive function, processing information and execution (orientation, attention, memory, naming objects, following simple tasks, and composing and writing down sentences). The total score ranges from 0 to 30 points, with 0 points indicating severe cognitive impairment. Cognitive assessment was conducted by trained psychological therapists. Functional status of the patients was assessed by B-ADL (15), a proxy assessment of functional limitations in the performance of everyday activities, conducted by occupational therapists. It contains 25 questions answered via a 10-point rating scale, with 0 meaning “never occurs” and 10 meaning “always occurs.” The total score ranges from 0 to 10 points, with 10 points indicating severe deficits in everyday life and dependency.

### Outcomes

The primary outcome of this study is the mortality rate. Mortality analysis will address overall survival of the whole sample as well as s subgroup analysis concerning dementia or DSD diagnosis. As secondary outcomes, readmissions to the department of geriatric psychiatry and discharge destination within 3 years after discharge were defined. All outcomes were assessed by trained psychiatric specialists (HS, FW) in a structured telephone interview. Three telephone contact attempts were made on different days. When unable to contact patients or caregivers, the subject was excluded from this study. These phone calls served to gain information about health condition, living status and readmission rates. In the case that a patient answered the call personally, health status was recorded as “alive.” When talking to a caregiver or legal guardian, they were asked if the patient is still alive or has passed away, also asking about the day of death where appropriate. Furthermore, information about care level as well as readmissions since discharge from the hospital were recorded. Care level was assessed on a zero- to three-point scale with three points meaning severe care dependency. The care level assessment by German nursing care insurance is included in the German social insurance system (16). Readmission was recorded by the answer to a “yes” or “no” question asking whether a readmission to the department of geriatric psychiatry within the 3 years occurred. These collected follow-up data were analyzed in relation to the patient's characteristics.

### Statistical Analysis

Data was analyzed using the IBM Statistical Package for the Social Sciences (SPSS) 25 for Windows (IBM Corporation, Route, Somers, NY, USA). A descriptive analysis of demographic and clinical characteristics was performed using incidences and proportions, means and standard deviations, as well as medians and interquartile ranges. Categorical variables were compared using the Chi-square test or Fisher's exact test, respectively. Continuous variables were compared using one-way ANOVA or the Kruskal–Wallis test, respectively. We defined the date of discharge as time zero for the survival analyses. Kaplan–Meier analysis were used to present 3 and 6 month survival as well as 1, 2, and 3 year survival according to dementia and DSD diagnosis, and log-rank tests were used to compare the groups. The association between discharge and time to death was analyzed using Cox proportional hazard models relating to the following preselected covariates: age, sex, BMI, MMSE, B-ADL, diagnosis, level of care, living situation at admission, living situation after discharge and days of hospitalization. All statistical tests were two-tailed, and an alpha error of up to 5% was applied.

## Results

**Figure 1:**
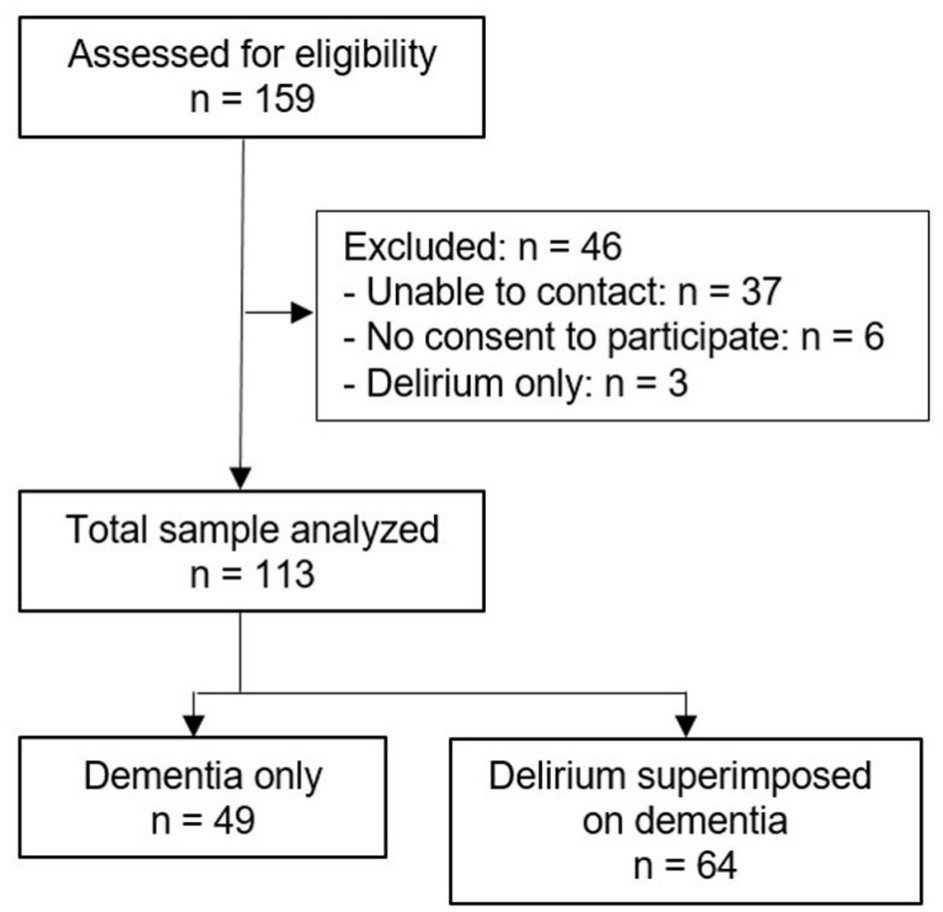
Study flow chart.

**Figure 2:**
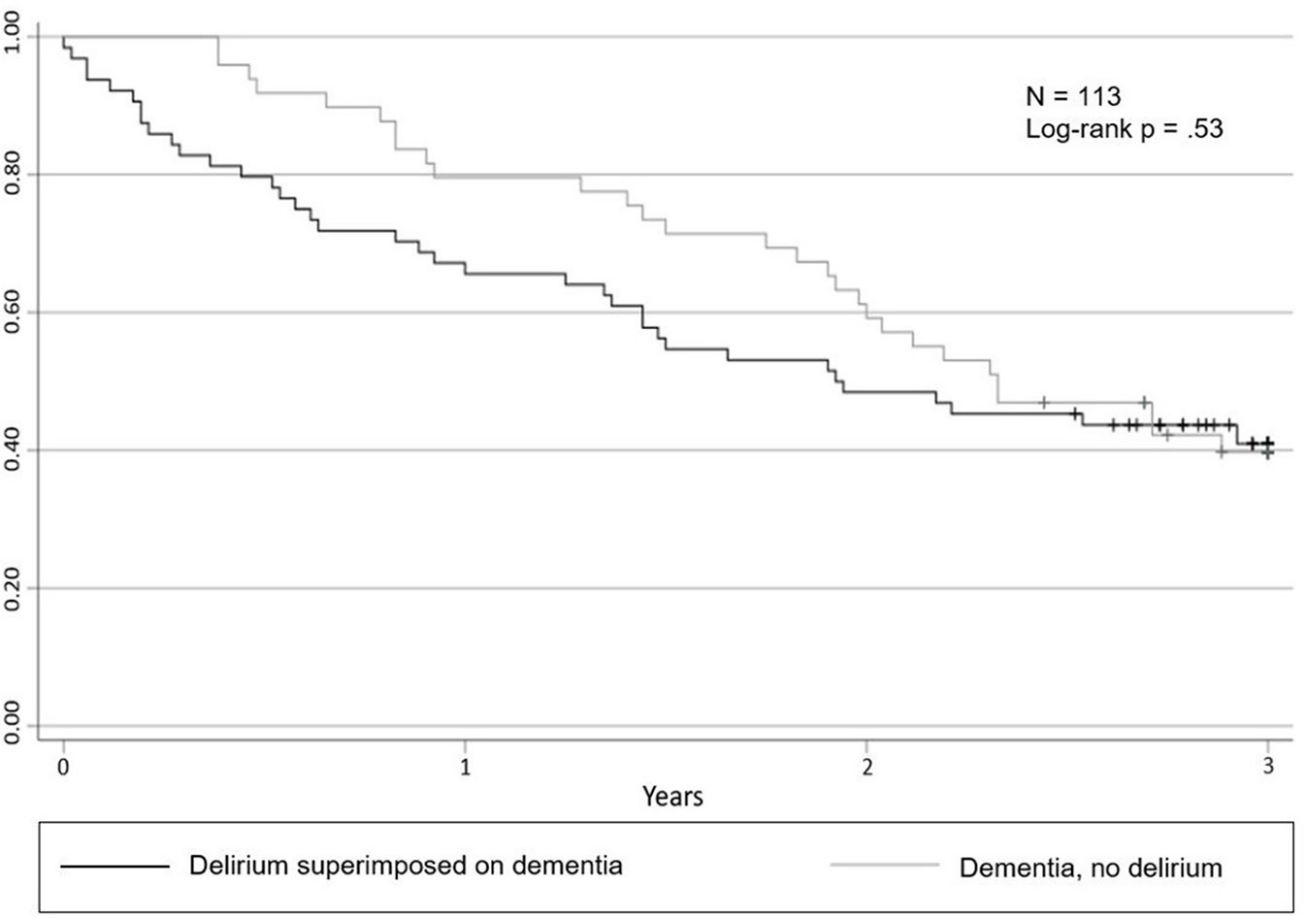
Probability of survival according to delirium and dementia diagnosis. Kaplan-Meier estimates representing the probability of survival after a 3 year observational period according to delirium and dementia diagnosis, with a corresponding log-rank group comparison.

In this study, 159 patients with dementia or DSD were screened for eligibility. In total, 46 patients (29%) were not eligible for inclusion (Figure 1). Sample characteristics (Table 1) showed no significant differences between the two groups (Dementia only and DSD) in analyzed mean values. Significant differences occurred in the distribution of subgroups regarding age, B-ADL, dementia diagnosis, hospital stay and living status at admission (*p* < 0.05). All sample characteristics and statistical group differences are listed in Table 1. The aftercare analysis revealed more discharges to nursing homes (Dementia: *n* = 16, 33%, *p* < 0.01; DSD: *n* = 24, 37%, *p* < 0.01) and less discharges to hospitals (Dementia: *n* = 10, 20%, *p* < 0.01; DSD: *n* = 23, 36%, *p* < 0.01) in both groups. In the first 3 years after discharge, 17 patients were readmitted to the department of geriatric psychiatry, with no significant differences between the two groups. In total, 66 (58%) patients died within the first 3 years after discharge from acute dementia care. The 3 year mortality for patients with dementia only was 59% (*n* = 29) and 58% (*n* = 37) for patients with DSD, revealing no significant difference (95% CI 91.9–112.5; log-rank test, *p* = 0.53) (Figure 2). The DSD group showed a significantly higher mortality rate during the first 3 months after discharge, with 14% (*n* = 9) vs. 0% from the dementia only group (95% CI 11.3–12.7; log-rank test, *p* = 0.01). The analysis revealed no significant differences in mortality between the groups after 6 months (8% DSD, 20% dementia only; *p* = 0.06), 1 year (34% DSD, 20% dementia only; *p* = 0.07) and 2 years (52% DSD, 41% dementia only; *p* = 0.13). Compared to the dementia only group, DSD patients showed higher hazard ratios for mortality at 3, 6 months, 1, 2, and 3 years (Table 2). The highest hazard ratios related to covariates were detected for vascular dementia (HR = 1.55, 95% CI = 0.78–3.08, *p* = 0.21), admission from homelessness (HR = 4.54, 95% CI = 0.60–34.58, *p* = 0.29) and discharge into nursing homes (HR = 1.27, 95% CI = 0.97–1.65, *p* = 0.08).

**Table 1:** Characteristics according to delirium and dementia diagnosis.

	**Dementia (*****n*** **=** **49)**			**DSD (*****n*** **=** **64)**			***p*-value**
	***n* (%)^a^**	**M^b^**	**SD**	***n* (%)**	**M**	**SD**	
Age (years)		80	5.7		82	6.7	0.29
Female	24 (49)			30 (47)			0.82
Dementia diagnosis				63 (98)			
Alzheimer's	17 (35)			35 (55)			0.04^c^
Vascular	5 (10)			13 (20)			0.15
Mixed type	27 (55)			16 (25)			0.01^c^
BMI (kg/m^2^)		26.4	3.7	58 (91)	25.2	4.6	0.13
MMSE^d^		18	4.6	45 (70)	18	5.3	0.55
B-ADL^e^		8	2.0	29 (45)	8	1.6	0.06
Care level at admission^f^	44 (90)	1	1.0	38 (59)	1	1.0	
Hospital days		35.8	19.4		36.0	25.0	0.96
Living status admission							
Homeless	0 (0)			1 (2)			0.38
Nursing home	10 (21)			9 (14)			0.37
Domesticity	28 (57)			24 (38)			0.04^c^
Hospital	11 (22)			30 (46)			0.01^c^
Living status discharge	47 (96)			60 (94)			
Nursing home	26 (55)			33 (55)			0.97
Domesticity	20 (43)			20 (33)			0.34
Hospital	1 (2)			7 (12)			0.06
Readmission				57 (89)			
Yes	7 (14)			10 (17)			0.05
No	42 (86)			47 (73)			0.05

a *assuming complete sample size unless otherwise indicated*.

b *patient's characteristics are presented as mean (M) and standard deviation (SD) for continuous variables and median and interquartile range for ordinal data*.

c *statistically significant differences*.

d *range 0–30 points (0 meaning severe symptoms)*.

e *range 0–10 points (10 meaning more severe deficits)*.

f *range 0–3 (3 meaning severe limitations and high dependency). B-ADL, Bayer activities of daily living scale; BMI, body mass index; DSD, delirium superimposed on dementia; MMSE, Mini-Mental State Examination*.

**Table 2:** Association between diagnosis and mortality (*n* = 113 patients).

**Diagnosis**	**Mortality, *n* (%)**	**Bivariate hazard ratio** **(95% CI; *p*-value)**
**3 months**		
Dementia only	0 (0)	Ref.
DSD	9 (14)	54.11 (0.24–12017.10; 0.15)
**6 months**		
Dementia only	4 (8)	Ref.
DSD	13 (20)	2.80 (0.91–8.60; 0.07)
**1 year**		
Dementia only	10 (20)	Ref.
DSD	22 (34)	1.96 (0.93–4.15; 0.07)
**2 years**		
Dementia only	20 (41)	Ref.
DSD	33 (52)	1.55 (0.88–2.73; 0.13)
**3 years**		
Dementia only	29 (59)	Ref.
DSD	37 (58)	1.17 (0.72–1.90; 0.54)

*CI, confidence interval; DSD, Delirium superimposed on dementia; Ref., reference category*.

## Discussion

The aim of this study was to investigate the mortality in a 3 year follow-up analysis after discharge from acute care of patients with dementia and DSD as well as readmissions and aftercare. The key results of this analysis revealed an overall 3 year mortality rate of 58% with significantly increased 3 month mortality in patients with DSD. Moreover, a readmission rate of 15% to the department of geriatric psychiatry was recorded and nursing homes were the predominant discharge destinations in both of our patient groups (55%). The overall mortality rates are similar to the results of existing studies conducting an up to 2 year follow-up analysis in comparable settings. Previous studies have described 53% mortality in the 2 years after treatment in hospital care (7) and a 1 year mortality of 50% in geriatric patients with delirium and 34% in patients without delirium (8). A possible reason for these overall high mortality rates in our sample could be the higher vulnerability of inpatients in geriatric psychiatry (17). In contrast, analysis of German mortality charts showed that the general geriatric population at the age of 80 is exposed to a 5.5% (male) and 3.5% (female) probability to die within the upcoming year (18). An important finding of our study is the prominent number of deaths 3 months after discharge; nine patients with DSD died within this period, but no patients with a dementia only diagnosis died in this short time after discharge. Other studies have also reported a higher risk of mortality in patients with DSD at 6 months and 1 year after discharge from acute care (7). The high risk of mortality for patients with DSD in the first 3 months is a particularly eminent finding of our investigation. This highlights the importance of close clinical monitoring, mainly within the first 3 months after hospital discharge, for patients with DSD, e.g., by general practitioners or in a hospital with regular examinations of vital functions, or potentially monitoring the patient's motor behavior (19). Moreover, this high short-term risk reflects the underlying causes of DSD, but could also be caused by serious consequences of physical restraints and urinary catheters, more falls and pressure ulcers, as well as sleep deprivation, acute malnutrition, dehydration and aspiration pneumonia in older patients with delirium (20). Within the 3 years of follow-up analysis, only a small number of patients (*n* = 17, 15%) were readmitted to the department of geriatric psychiatry. There are no investigations available to date that have reported or analyzed readmission rates in geriatric psychiatry. The potentially few readmissions could be due to the high mortality risk in patients with dementia or DSD (6). Concerning discharge destinations, an analysis from North-West England, where 83% of the patients (*n* = 82) were able to return to home after treatment in acute psychogeriatric wards (10), contrasts with the findings of this study. A possible explanation could be the high levels of functional dependencies in this sample (B-ADL score MED = 8, IQR = 1.9, with 10 points indicating high dependency), which has shown to be a key predictor of discharge destination in old age psychiatry (21).

### Limitations

The discussion of our results should consider the following key limitations. Although patients with delirium are prominent in geriatric care, the three patients (2%) with a delirium only diagnosis recruited in our sample were not considered in the further analysis. As these three patients were the only patients that did not have a dementia diagnosis, we decided to excluded them to prevent possible bias in the analysis. This could be due to the specialization of the LVR Hospital on the treatment of patients with dementia and DSD. Moreover, a subgroup of 37 patients (23%) were excluded as they could not be contacted in the telephone calls. These patients did not differ in diagnosis or key sociodemographic features at baseline, still we cannot prevent a possible bias caused by this. This issue can be addressed by using civil registries in future investigations.

### Conclusion

This is the first study applying a 3 year follow-up analysis on the mortality rates, readmissions and aftercare institutions in patients with dementia and delirium. Further planned studies should address mortality in relation to the discharge destinations and other long-term effects in acute mental health care (10, 17). Larger multi-center studies, conducting civil registry-based analyses could provide more relevant insights in this field of research and healthcare. The enhancement of psychogeriatric expertise in the outpatient setting in order to prevent DSD beforehand, thus preventing hospital admissions and mortality, seems critical to us. The key results of this study indicate an overall high mortality rate of both DSD and dementia only patients within 3 years after hospital discharge. Patients with DSD especially face a high short-term risk of mortality within 3 months after discharge. These findings serve as an important call for increased efforts to improve research and treatment of delirium and dementia in clinical practice (6, 10). More insights into the long-term effects of acute mental health care will help to improve the state-of-art treatment and discharge management in acute geriatric care.

## Data Availability Statement

The raw data supporting the conclusions of this article will be made available by the authors, without undue reservation.

## Ethics Statement

The studies involving human participants were reviewed and approved by Ethics Committee of the North Rhine Medical Association Chamber (Reference Number 2018192). The patients/participants provided their written informed consent to participate in this study.

## Author Contributions

TS and TF co-formulated the research question, co-designed the study, co-conducted data extractions, led on data analysing, and drafting the article prior to submission. HS and FW led on data extraction. IR, RT, and SS provided clinical, methodology and analysis advice, and contributed to data interpretation. WZ and PH made substantial contributions to the conception and design of the work, provided methodology, and analysis advice. All authors contributed to the article and approved the submitted version.

## Funding

This study was funded by the author's institutional budgets. This funding did not influence the scientific investigation and reporting.

## Conflict of Interest

SS is an employee by KMG Kliniken. The remaining authors declare that the research was conducted in the absence of any commercial or financial relationships that could be construed as a potential conflict of interest.

## Publisher's Note

All claims expressed in this article are solely those of the authors and do not necessarily represent those of their affiliated organizations, or those of the publisher, the editors and the reviewers. Any product that may be evaluated in this article, or claim that may be made by its manufacturer, is not guaranteed or endorsed by the publisher.
